# Conditioning with fludarabine and treosulfan compared to FLAMSA-RIC in allogeneic stem cell transplantation for myeloid malignancies: a retrospective single-center analysis

**DOI:** 10.1007/s00277-022-04822-x

**Published:** 2022-04-01

**Authors:** Krischan Braitsch, Alix Schwarz, Katrin Koch, Mara Hubbuch, Helge Menzel, Ulrich Keller, Katharina S. Götze, Florian Bassermann, Peter Herhaus, Mareike Verbeek

**Affiliations:** 1 Internal Medicine III, Hematology and Medical Oncology, School of Medicine, Technische Universität München, Ismaninger Strasse 22, 81675 Munich, Germany; 2 Medical Department I, Malteser Krankenhaus St. Franziskus-Hospital, Waldstraße 17, 24939 Flensburg, Germany; 3 German Cancer Consortium (DKTK) and German Cancer Research Center (DKFZ), Im Neuenheimer Feld 280, 69120 Heidelberg, Germany

**Keywords:** Fludarabine/treosulfan, FLAMSA-RIC, Allogeneic transplantation, Conditioning regimen

## Abstract

Reduced intensity conditioning (RIC) and reduced toxicity conditioning (RTC) regimens enable allogeneic hematopoietic stem cell transplantation (alloSCT) to more patients due to reduction in transplant-related mortality (TRM). The conditioning regimens with fludarabine and treosulfan (Flu/Treo) or fludarabine, amsacrine, cytarabine (FLAMSA)-RIC have shown their efficacy and tolerability in various malignancies. So far, no prospective study comparing the two regimens is available. Two studies compared the regimens retrospectively, in which both provided similar outcome. In this retrospective, single-center analysis, these two regimens were compared with regard to outcome, rate of acute and chronic graft versus host disease (GvHD), and engraftment. 113 consecutive patients with myeloid malignancies who received Flu/Treo or FLAMSA-RIC conditioning prior to alloSCT between 2007 and 2019 were included. Except for age, previous therapies, and remission status before alloSCT, patient characteristics were well balanced. The median follow-up time within this analysis was 44 months. There was no significant difference in absolute neutrophil count (ANC) or platelet engraftment between the two conditioning regimens. Overall survival (OS), the relapse-free survival (RFS), and the TRM were not significantly different between the two cohorts. The rate of GvHD did not differ between the two groups. In summary, this retrospective analysis shows that there is no major difference regarding tolerability and survival between the Flu/Treo and FLAMSA-RIC regimens. Despite several limitations due to uneven distribution concerning age and remission status, we demonstrate that Flu/Treo and FLAMSA-RIC provide similar outcomes and are feasible in older and intensively pre-treated patients.

## Introduction

Allogeneic hematopoietic stem cell transplantation (alloSCT) is a potentially curative treatment for hematological malignancies such as acute myeloid leukemia (AML), myelodysplastic syndromes (MDS), and myeloproliferative neoplasms (MPN). The intensity of the conditioning regimen is known to have a significant impact on the outcome and success of transplantation and the choice of regimen in the different disease settings has been discussed and reviewed intensely in recent years [1, 2]. However, conditioning regimens are heterogeneously used and there is still a lack of knowledge in terms of direct comparison between the multiple available protocols.

Myeloablative conditioning regimens (MAC) based traditionally on total body irradiation (TBI) or busulfan may be accompanied by serious short- and long-term side effects resulting in relatively high TRM and organ toxicity rates. As the majority of patients with AML, MDS, or MPN are older than 50 years and often present with comorbidities, reduced toxicity conditioning (RTC) or reduced intensity conditioning (RIC) regimens have been increasingly used as an alternative approach to allow curative alloSCT in patients otherwise not eligible. However, RIC regimens have shown limitations compared with conventional regimens, e.g., increased relapse and TRM rates [3–6]. Furthermore, a significant survival advantage for MAC though at the expense of expected high TRM rates has recently been demonstrated in a prospective randomized study for AML and MDS patients eligible for MAC and RIC [7]. Therefore, it remains difficult to determine the optimal conditioning protocol for each individual patient.

Other factors such as remission status prior to alloSCT, previous therapies, or disease entity, as well as individual risk profiles such as comorbidities and age, also have a crucial impact on outcome. To address these issues, various conditioning regimens, both RIC and MAC, for different patient settings have been introduced over the last years. The fludarabine, amsacrine, cytarabine-RIC (FLAMSA-RIC) regimen was initially described in 2005 as an effective and well-tolerated sequential conditioning approach for AML and MDS patients with refractory or progressive disease with an otherwise dismal prognosis [8]. Meanwhile, this regimen is well established and broadly implemented in other disease settings as well. The original combination of chemotherapy and TBI is often modified by replacing TBI with busulfan and more recently also with treosulfan, thereby further improving the tolerability [9]. Treosulfan in combination with fludarabine (Flu/Treo) was introduced in 2004 as a conditioning regimen with an excellent toxicity profile in various disease entities and has continuously gained utilization ever since [5, 10–16].

Recently, several trials have pointed towards favorable aspects of treosulfan, showing remarkable effectiveness, good feasibility, and advantages in terms of survival compared to conventional busulfan-containing conditioning regimens. Interestingly, the benefit could also be shown in elderly patients even though treosulfan-containing conditioning regimens were revealed and considered as being more of MAC than RIC character [17, 18].

As to date, no prospective studies comparing the two conditioning regimens exist. Two retrospective analyses showed no significant difference in terms of survival between the two conditioning regimens [14, 15]. While patients in the FLAMSA-RIC group had a lower risk of relapse and superior leukemia-free survival, they also had increased rates of GvHD [15]. In relapsed or refractory AML patients, no significant difference with regard to outcome could be observed [14].

We performed a retrospective analysis of patients with AML, MDS, and MPN that received either Flu/Treo or FLAMSA-RIC before alloSCT between 2007 and 2019.

## Patients and methods

### Patients

This retrospective analysis included 113 consecutive patients with hematologic malignancies receiving Flu/Treo or FLAMSA-RIC conditioning before alloSCT at the university hospital of the Technische Universität München between 2007 and 2019. Patient data concerning outcome, engraftment, and GvHD were analyzed retrospectively by medical chart review. All patients that received at least one dose of chemotherapy of Flu/Treo or FLAMSA-RIC conditioning were included. Missing data were accepted due to the retrospective nature of this study. Patients were eligible for alloSCT based on their underlying malignancy and institutional guidelines. All patients gave written consent according to local center guidelines.

Disease stage prior to alloSCT was defined according to the EBMT risk score [19]. Cytogenetic and molecular genetic risk was allocated in good, intermediate, and high risk. This classification was according to ELN 2017 genetic risk stratification for AML and IPSS for MDS [20, 21]. MPN were allocated to the high-risk group by absence of the bcr-abl protein or presence of the T315I mutation within the tyrosine kinase domain of bcr-abl.

HLA typing was based on high-resolution typing of MHC class I and class II. An HLA-identical related donor was defined as an 8/8 compatible donor. An HLA-identical unrelated donor was defined as 10/10 compatible. Mismatched related or unrelated donor was defined as one allele or antigen mismatch. Cord blood grafts that the one patient with dual umbilical cord blood transplantation received were 6/6 matched.

Previous therapies were extracted from medical charts and intensive chemotherapy such as induction, or consolidation therapy was distinguished from treatment with hypomethylating agents (HMA). Each applied cycle of chemotherapy was counted to reflect the cumulative amount of previous treatment. Cytoreductive therapy with hydroxycarbamide was not counted as a separate line of therapy.

### Conditioning regimens

The conditioning regimen with Flu/Treo consisted of fludarabine 30 mg/m^2^ from day − 7 to day − 3 and treosulfan 14 g/m^2^ in patients < 60 years or 12 g/m^2^ in patients > 60 years old from day − 7 to day − 5 [10]. Conditioning according to the FLAMSA-RIC protocol was conducted with a modification to the RIC-part as previously described [8]. In the modified FLAMSA-RIC protocol, TBI was substituted by busulfan. Briefly, patients received fludarabine 30 mg/m^2^, cytarabine 2 g/m^2^, and amsacrine 100 mg/m^2^ from day − 12 to day − 9 followed by busulfan 0.8 mg 4 times daily from day − 5 to day − 4 and cyclophosphamide 60 mg/kg from day − 3 to day − 2.

Initial immunosuppression was identical in both conditioning regimens and included a calcineurin- inhibitor (cyclosporine A (CSA) or Tacrolimus (TAC)) and mycophenolate mofetil (MMF).

### Evaluation of response

Engraftment of WBC and platelets was defined as the first of three days with an ANC count > 0.5 × 10^9^/l and the first of seven days with an untransfused platelet count > 20 × 10^9^/l. For the grading of acute and chronic GvHD, the National Institutes of Health consensus criteria were used [22]. Fluorescence in situ hybridization (FISH) analysis for X and Y chromosomes were used for chimerism analysis in sex-mismatch transplantations. For sex-matched transplantation, the chimerism was evaluated using PCR analysis of microsatellite markers. Determination of chimerism, response, and relapse was performed on bone marrow aspirates.

### Statistical analysis

All statistical tests were performed using GraphPad Prism (GraphPad Software). *P*-values < 0.05 were considered statistically significant. Comparisons of related metric measurements were performed using student’s *t*-test or Fisher’s exact test if used to compare quantitative data between two independent samples. Kaplan–Meier-plots were calculated using log-rank (Mantel-Cox) test.

Due to the retrospective analysis, sample size was chosen based on the number of consecutive patients treated with the selected conditioning regimens in the specified time frame.

## Results

### Patient and donor characteristics

In this analysis, 113 patients who were treated with alloSCT after receiving a RTC regimen either with Flu/Treo or FLAMSA-RIC between October 2007 and July 2019 were retrospectively analyzed. Seventy-two received conditioning with Flu/Treo, and 41 received FLAMSA-RIC. Both regimens were used concurrently throughout the analyzed time frame. The median age was 61 years (range 41–71 years) in the Flu/Treo group and 55 years (range 41–74 years) in the FLAMSA-RIC group. Fifty-seven percent of patients in the Flu/Treo group were over 60, contrary to only 39% of patients in the FLAMSA-RIC group. The patient characteristics concerning disease, cytogenetic, and molecular genetic risk were well balanced between the two groups (Table 1).

**Table 1:** Patient characteristics of the Flu/Treo and FLAMSA-RIC cohorts. Abbreviations: Flu/Treo: fludarabine/treosulfan; FLAMSA-RIC: fludarabine, cytarabine, amsacrine – reduced intensity conditioning; AML: acute myeloid leukemia; MDS: myelodysplastic syndrome; MPN: myeloproliferative neoplasm; tAML: therapy-related AML; n.a.: not annotated; HMA: hypomethylating agents; CR: complete remission; CRi: CR with incomplete hematologic recovery

	Flu/Treo	FLAMSA-RIC
Number of patients	72	41
Age, years		
Median (range)	61 (41–71)	55 (41–74)
> 60 years	40 (57%)	16 (39%)
Sex		
Male	44 (61%)	17 (41%)
Female	28 (39%)	24 (59%)
Disease		
De novo AML	27 (37%)	16 (39%)
Secondary AML	20 (28%)	19 (46%)
MDS	18 (25%)	3 (7%)
Other (MDS/MPN; tMDS, tAML)	7 (10%)	3 (7%)
ELN-Risk criteria for AML patients		
Favorable risk	6 (12%)	4 (11%)
Intermediate risk	18 (37%)	12 (33%)
Adverse risk	22 (45%)	17 (47%)
n.a./missing	3 (6%)	3 (8%)
Previous therapies		
Intensive chemotherapy	44 (61%)	25 (61%)
HMA monotherapy	19 (26%)	11 (27%)
Number of intensive chemotherapies	3 (1–7)	2 (1–6)
Remission before transplantation		
1st CR/CRi	31 (43%)	9 (22%)
2nd CR/CRi	6 (8%)	0 (0%)
Partial remission	10 (14%)	6 (15%)
Stable disease	14 (20%)	7 (17%)
Relapsed	2 (3%)	4 (10%)
Refractory	2 (3%)	5 (12%)
Upfront	6 (8%)	3 (7%)
n/a	1 (1%)	1 (2%)
Progress to HMA monotherapy	0 (0%)	6 (15%)

Forty-four (61%) patients in the Flu/Treo group had previously received intensive induction or consolidation therapy and 19 (26%) were treated with HMA alone. In the FLAMSA-RIC group, 25 patients (61%) had received intensive chemotherapy, and 11 (27%) were treated with HMA monotherapy. In median, patients in the Flu/Treo cohort had received more cycles of chemotherapy compared to patients in the FLAMSA-RIC group (Flu/Treo *n* = 3 (1–7); FLAMSA-RIC *n* = 2 (1–6), *p* = 0.01). Thirty-one (43%) were in first complete remission (CR) or CR with incomplete hematologic recovery (CRi) in the Flu/Treo group in contrast to only 9 (22%) in the FLAMSA-RIC group. Six (8%) patients achieved a second CR before alloSCT in the Flu/Treo group, and 6 (15%) patients in the FLAMSA-RIC group had MDS and progressed to sAML during HMA treatment. More patients were either relapsed or refractory in the FLAMSA-RIC group (22%) compared to the Flu/Treo cohort (6%).

In both groups, the most frequent stem cell source was peripheral blood (PB). In the FLAMSA-RIC group all but six patients received PB-derived hematopoietic stem cells. Of the six patients in the FLAMSA-RIC cohort, 5 received cord blood (CB) grafts and 1 received combined a combined PB/bone marrow (BM) graft. In the Flu/Treo group 67 (93%) patients received PB, 4 (6%) received BM, and data was unavailable for one patient. There was no significant difference between the administered doses of CD34 + cells/kg body weight (BW) (Flu/Treo vs. FLAMSA-RIC, median range: 6.8 (1.6–12.6) vs. 7.5 (1.8–17); *p* = 0.13; Table 2).

**Table 2:** Transplant characteristics of the Flu/Treo and FLAMSA-RIC cohorts. PB: peripheral blood; BM: bone marrow; BW: body weight; ATG: anti-thymocyte globuline; HLA: human leukocyte antigen; CMV: cytomegalovirus; ANC: absolute neutrophil count

	Flu/Treo	FLAMSA-RIC
Number of patients	72	41
Graft source		
PB	67 (93%)	35 (85%)
BM	4 (6%)	0 (0%)
Combined PB/BM	0 (0%)	1 (2%)
Cord blood	0 (0%)	5 (12%)
CD34 + cells/kg BW	6.8 (1.6–12.6)	7.5 (1.8–17)
Immunosuppression		
ATG	68/70 (96%)	41/41 (100%)
Donor characteristics		
Donor type		
HLA-identical sibling	21 (29%)	13 (32%)
HLA-identical unrelated	49 (68%)	28 (68%)
mismatched unrelated	2 (3%)	0 (0%)
CMV		
High risk	15/71 (21%)	6/38 (16%)
Engraftment		
ANC engraftment		
No. of patients	71/72 (99%)	37/41 (90%)
Days, median (range)	21 (10–35)	23 (12–48)
Platelet engraftment		
No. of patients	69/72 (96%)	37/41 (95%)
Days, median (range)	20 (10–35)	20 (9–42)

Per institutional guidelines, there was no difference between the initial immunosuppression as all patients received calcineurin inhibitors and MMF. The proportion of patients receiving ATG was not different between the two groups (Flu/Treo vs. FLAMSA-RIC: 68 (96%), vs. 41 (100%); Table 2). Data was unavailable for two patients in the Flu/Treo group.

In both cohorts, most patients had an HLA-identical unrelated donor (Flu/Treo vs. FLAMSA-RIC: 49 (68%) vs. 28 (68%)). Two patients (3%) had mismatched unrelated donors in the Flu/Treo cohort vs. no mismatched unrelated donors in the FLAMSA-RIC group. There were slightly more HLA-identical sibling donors within the FLAMSA-RIC group (Flu/Treo vs. FLAMSA-RIC: 21 (29%) vs. 13 (32%); Table 2). The median follow-up time was 44 months among all patients.

### Engraftment

All together 108 (96%) patients reached primary ANC engraftment (Flu/Treo vs. FLAMSA-RIC, no. of patients: 71/72 vs. 37/41) and 106 (94%) patients showed platelet engraftment (Flu/Treo vs. FLAMSA-RIC: 69/72 vs. 37/41). The ANC engraftment was achieved in median 21 (range 10–35) days after transplantation in the Flu/Treo group and in median 23 (range 12–48) in the FLAMSA-RIC group. The thrombocyte engraftment occurred in median 20 days (range 10–35) after transplantation in the Flu/Treo group and in median 20 days after transplantation in the FLAMSA-RIC group (range 9–42). There was neither a significant difference in ANC (*p* = 0.28) nor in thrombocyte (*p* = 0.90) engraftment within the two groups. Data are shown in Table 2.

### Outcome

**Fig. 1:**
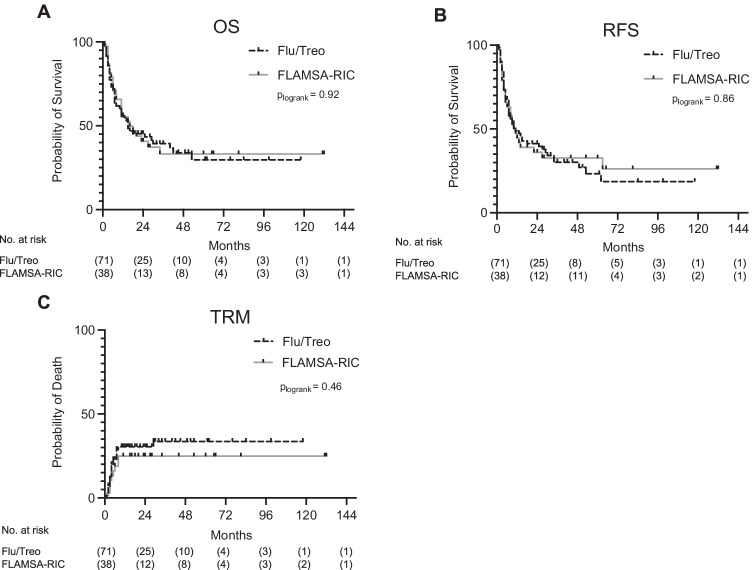
Outcomes of the Flu/Treo and FLAMSA-RIC cohorts. **A** Overall survival, **B** relapse-free survival (RFS), and **C** transplant-related mortality (TRM). Abbreviations: Flu/Treo fludarabine/treosulfan; FLAMSA-RIC fludarabine, cytarabine, amsacrine – reduced intensity conditioning; no. number

There was no significant difference in median OS with 15 months in the Flu/Treo group compared to 16 months in the FLAMSA-RIC group (*p* = 0.92) (Fig. 1A). The 1-year, 2-year, and 5-year OS rates in the Flu/Treo compared to the FLAMSA-RIC groups were 56% and 55%, 45% and 41%, and 30% and 33%, respectively. At the median follow-up time of 44 months, 34% of patients were alive in Flu/Treo group compared to 33% alive in the FLAMSA-RIC group. Further, no significant difference in OS could be observed among patients aged < 60 years (*p* = 0.92) or aged ≥ 60 years (*p* = 0.98) among all patients. Per institutional guidelines, the cumulative dosage of treosulfan in the Flu/Treo group was 36 g/m^2^ for patients aged ≥ 60 years and 42 g/m^2^ for patients aged < 60 years. These different dosages had no significant impact on OS (*p* = 0.55). There was also no significant difference in OS in the Flu/Treo (*p* = 0.77) and the FLAMSA-RIC group (*p* = 0.98) for patients with AML, MDS, or MDS/MPN. Similarly, no significant differences concerning OS could be observed between patients with sAML and de novo AML in both cohorts.

The median RFS was also similar in both groups with 11 months in the Flu/Treo cohort vs. 10.5 months in the FLAMSA-RIC group (*p* = 0.86) (Fig. 1B). Age of 60 years or older (FLAMSA-RIC, *p* = 0.68; Flu/Treo, *p* = 0,21) and type of disease (FLAMSA-RIC, *p* = 0.91; Flu/Treo, *p* = 0.87) had no significant influence on RFS, in both groups. The TRM was 31% among all patients. In the Flu/Treo group, the TRM was 32%, and 29% in FLAMSA-RIC group with no significant difference (*p* = 0.45). Most deaths not related to relapse were caused either by uncontrolled GvHD (4 patients in the Flu/Treo group and 1 patient in the FLAMSA-RIC group) or infections (16 patients in the Flu/Treo group and 11 patients in the FLAMSA-RIC group). One patient in the Flu/Treo group died of cardiac arrest.

A total of 12 (29%) patients received donor lymphocyte infusions (DLI) in the FLAMSA-RIC group, compared to 6 (8%) patients in the Flu/Treo group. Among the 12 patients receiving FLAMSA-RIC conditioning, 5 received prophylactic DLI according to Schmidt et al. [8]. The remaining 7 patients had either hematological relapse (*n* = 4), molecular relapse (*n* = 1), or loss of donor chimerism (*n* = 2). In the Flu/Treo group, no prophylactic DLI were applied; 4 patients received DLI for hematological relapse and 2 for molecular relapse.

### GvHD

**Fig. 2:**
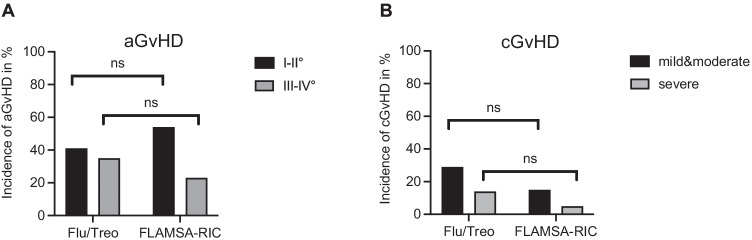
Incidence of acute and chronic GvHD of the Flu/Treo and FLAMSA-RIC cohorts. **A** acute GvHD I–II° and acute GvHD III–IV°; **B** chronic GvHD (mild, moderate) and chronic GvHD (severe) for both groups respectively. Abbreviations: Flu/Treo fludarabine/treosulfan; FLAMSA-RIC fludarabine, cytarabine, amsacrine – reduced intensity conditioning; no. number; GvHD graft versus host disease; ns not significant

Out of the 113 studied patients, a total of 84 (76%) developed an acute GvHD and data was unavailable for 3 patients. Rates of acute GvHD I/II were slightly higher in the FLAMSA-RIC group (54%) compared to the Flu/Treo group (40%) but no significant difference could be observed (*p* = 0.23). Respectively, acute GvHD III/IV° occurred more frequently in the Flu/Treo cohort (35%) compared to the FLAMSA-RIC group (23%) without statistical significance (*p* = 0.20) (Fig. 2A).

Thirty-seven (33%) patients developed chronic GvHD; data was unavailable for 4 patients. Occurrence of mild or moderate cGvHD did not differ significantly in patients in the Flu/Treo group (27%) compared to the FLAMSA-RIC cohort (15%) (*p* = 0.16). Rates of severe cGvHD were also observed more frequently with 15% in the Flu/Treo group vs. 5% in the FLAMSA-RIC group, however there was no significant difference either (*p* = 0.20) (Fig. 2B).

## Discussion

AlloSCT represents a curative treatment option in patients with unfavorable MDS or MPN and primary or relapsed AML. These diseases occur with higher incidence in the older population, where regimens with a balanced toxicity and tolerability profile as compared to standard MAC regimens are often required. The RTC regimens with Flu/Treo and FLAMSA-RIC have been developed and elaborated in older patients with myeloid malignancies. In this retrospective, single-center analysis, we show that the RTC regimens with Flu/Treo and FLAMSA-RIC are feasible conditioning regimens for older patients and do not significantly differ concerning engraftment, outcome, or the rate of GvHD.

Compared to MAC regimens, in particular, the low rate of NRM resulted in a comparable OS rate of RIC and RTC regimens. Seminal studies on the conditioning regimen with Flu/Treo have shown a NRM rate ranging between 20 and 35% [5, 10, 12, 23]. This is similar to the NRM rate of 32% seen within the Flu/Treo group in this analysis, considering the high median age and the high number of previous treatments in our cohort. Although there is no statistical significance concerning the NRM rate between the Flu/Treo and FLAMSA-RIC group, the NRM rate of 29% of the FLAMSA-RIC group was higher compared to a NRM rate of approximately 20% reported in the literature [8, 24, 25]. The high proportion of patients with advanced disease stage, active or refractory disease, and a high median age within the FLAMSA-RIC group in our analysis might partially explain this discrepancy.

The 1-year and 2-year OS rate was 56% and 39% for the Flu/Treo and 45% and 41% for the FLAMSA-RIC group, respectively. These results compare less favorable to the seminal study on the Flu/Treo protocol from Casper et al. with an OS rate of 73% at a median follow-up of 22 months. However, patients in this study were, with a median age of 49 years, younger compared to the population in this retrospective analysis [10]. A slightly lower 2-year OS rate of 34% after conditioning with Flu/Treo was seen in a study conducted in older patients with sAML and MDS [5]. The OS rate of the FLAMSA-RIC group compares well to a 2-year OS rate of 40% in the study of Schmid et al. [8]. Sheth et. al reported a higher OS and RFS rate in their retrospective study [15]. However, patients in the Flu/Treo group were in median 57 years old compared to 61 years in our study and patients in the large cohort of Sheth et al. exclusively had AML and were in their first (86% and 79%) or second (14% and 21%) CR in both the Flu/Treo and FLAMSA-RIC group, respectively. Further, that study included less than one-third of the patients with adverse risk according to 2017 ELN criteria and 79% and 78% had de novo AML compared to 37% and 39% in our study. Another study published by Saraceni et al. compared these two conditioning regimens in patients with relapsed or refractory AML and showed an OS 37% for Flu/Treo and 34% for FLAMSA-RIC conditioning [14]. In line with the other study, patients were younger compared to our cohort with a median age of 53 years in both groups.

Patients from our study had received up to 7 (ranging from 1 to 7, in median 3 in the Flu/Treo group vs. 2 in the FLAMSA-RIC group) cycles of intensive chemotherapy. This parameter is not available in most retrospective studies. However, this might be an additional factor explaining the lower rates of OS and RFS.

The overall rate of acute GvHD in the Flu/Treo group of 76% was high compared to previously reported rates ranging from 46 to 62% [5, 10, 26]. The rate of acute GvHD in the FLAMSA-RIC cohort of 77% compared similarly to a rate between 53 and 74% described in the seminal studies [8, 9, 27, 28]. The rates of chronic GvHD of 41% in the Flu/Treo group and 20% in the FLAMSA-RIC group were not excessive compared to the aforementioned studies. Exposure to multiple lines of chemotherapy prior to alloSCT has been associated with higher rates of acute GvHD and lower GvHD- and relapse-free survival [29, 30]. This might partially explain the comparatively high rate of acute GvHD observed in the Flu/Treo group. Although the results of this analysis compare well with the literature concerning outcome and GvHD rate, and although no significant differences between the two groups concerning engraftment, outcome, or higher grade acute and chronic GvHD were observed, there are obviously several limitations. First, mainly due to the different indications of the two conditioning regimens, Flu/Treo for older patients and FLAMSA-RIC for advanced/refractory diseases, the groups are not well-balanced concerning patient age and most importantly concerning remission prior to alloSCT. Patients within the Flu/Treo group were older with a median age of 61 years compared 55 years within the FLAMSA-RIC group. While patients within both groups (FLAMSA-RIC group (47%), Flu/Treo group (45%)) had an advanced disease stage, CR rates were higher in the FLAMSA-RIC group. Although age, disease stage, and remission status are independent risk factors for a worse outcome after alloSCT and are complementary distributed, this clearly limits the conclusions drawn from this retrospective comparison. Second, due to the retrospective analysis, data concerning exact time points of GvHD occurrence and GvHD management are limited.

While the FLAMSA-RIC protocol is a well-established regimen for patients with relapsed/refractory AML or patients with high-risk MDS, conditioning with Flu/Treo was likewise able to induce CR among patients with active myeloid malignancies [31]. Flu/Treo has as well been proven to be superior to a MAC regimen with TBI in patients with MDS [11].

This retrospective single-center analysis reflects a real-world patient population with intensive prior therapy before alloSCT and a high median patient age. In this cohort, we demonstrate comparable outcomes of the RTC regimens Flu/Treo and FLAMSA-RIC in patients with myeloid malignancies and show that both regimens are feasible. However, especially due to the aforementioned limitations, prospective randomized trials that directly compare those two conditioning regimens, especially in older patients with refractory/relapsed AML, are urgently needed.
